# Confinement Effects on the Benzene Orientational Structure

**DOI:** 10.1002/anie.201713115

**Published:** 2018-03-13

**Authors:** Marta Falkowska, Daniel T. Bowron, Haresh Manyar, Tristan G. A. Youngs, Christopher Hardacre

**Affiliations:** ^1^ School of Chemical Engineering and Analytical Science The University of Manchester UK; ^2^ ISIS Neutron and Muon Source Science and Technology Facilities Council UK; ^3^ School of Chemistry and Chemical Engineering Queen's University Belfast UK

**Keywords:** arenes, heterogeneous catalysis, liquids, mesoporous materials, neutron diffraction

## Abstract

Liquids under confinement exhibit different properties compared with their corresponding bulk phases, for example, miscibility, phase transitions, and diffusion. The underlying cause is the local ordering of molecules, which is usually only studied using pure simulation methods. Herein, we derive experimentally the structure of benzene confined in MCM‐41 using total neutron scattering measurements. The study reveals a layering of molecules across a pore, and four concentric cylindrical shells can be distinguished for a pore with the radius of 18 Å. The nanoscale confinement of the liquid has a major effect on the spatial and orientational correlations observed between the molecules, when compared with the structure of the bulk liquid. These differences are most marked for molecules in parallel configurations, and this suggests differences in chemical reactivity between the confined and bulk liquids.

Confined liquids are an important subject of research in many areas such as geology, biology, food, catalysis and drug preservation.^1^ Due to reduced dimensionality and large surface and interface effects, liquids that are subjected to some geometric constraints on the nanoscale can have different properties to those they display under standard bulk conditions.^2^ For instance, the local molecular density profile can show fluctuations, and the extent of layering of molecules across the pore is dependent on the pore radius.^3^ X‐ray and neutron scattering experiments on water confined in nanopores have demonstrated that liquid states can persist down to temperatures much lower than in the bulk liquid,^4^ and there is also recent evidence that confinement can induce microphase separation of binary liquid mixtures. Such phenomena have been reported for aqueous solutions of glycerol,^5^ and for *tert*‐butanol and toluene binary mixtures^6^ confined in MCM‐41, although these solutes are fully miscible under bulk solution conditions. Understanding the structure of confined liquids is, therefore, an important factor that can help explain why liquid properties change when molecular interactions are subject to nanoscale steric constraints.

Herein, wide *Q*‐range total neutron scattering has been used to obtain insights into the nature of benzene confined in the pores of a Pt/MCM‐41 catalyst, and which enables characterization of the system's structural aspects on multiple levels simultaneously in a single measurement (for more experimental details see the Supporting Information). These structural properties include, for example, local ordering of molecules in a sample and the orientation of molecules with respect to the confining interface or to other molecules. Benzene is the archetypical example of an aromatic compound and is frequently used as a simple model material to investigate π‐π interactions that are important in many complex processes such as nucleobase stacking within DNA and RNA molecules or in protein folding.^7^, ^8^ MCM‐41 is an idealized model catalyst support formed from a porous silica glass, comprising a hexagonally organized lattice of cylindrical pores. For the system under investigation, features corresponding to the regular ordering of the pore network can be observed via Bragg scattering peaks in the low‐*Q* range (corresponding to correlations at longer real‐space distances) of the total structure factor (Figure 1), whilst the diffuse scattering arising from the liquid present in the pores, from the atomic structure of the glassy catalyst support, and from interactions between the two can be seen in the higher *Q*‐range (corresponding to local molecular and atomic correlations over short real‐space distances).

**Figure 1:**
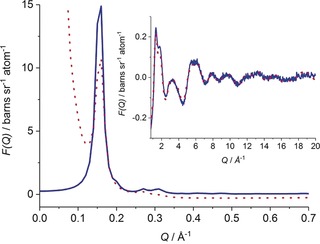
Total structure factor obtained for benzene‐*d_6_* confined in MCM‐41. Dotted line expresses the experimental data while the solid line shows that generated by Empirical Potential Structure Refinement (EPSR) following constraint by the experimental data. In the inset, the total structure factor over the higher *Q*‐range is enlarged for clarity.

Few structural studies on liquids in confinement based on scattering data have been reported, to date, and the majority of these focus only on either one of the low (small angle) or high *Q*‐range (diffuse scattering) scattering pattern regions.^1^, ^9^, ^10^, ^11^, ^12^, ^13^ In this study we have constructed an atomistic model which reproduces the whole total neutron scattering patterns over the continuous *Q*‐range 0.05≤*Q*≤20 Å^−1^ using Empirical Potential Structure Refinement (EPSR)^14^, that is, an atomistic Monte Carlo simulation that is constrained by the experimental total neutron scattering data. It should be noted that as total neutron scattering supplies only static structural information on the sample, no information on dynamics is obtained from EPSR modelling. The procedure introduced in a study on water confined in MCM‐41^15^ and applied in a study of structuring of N_2_ in MCM‐41 was followed.^16^

As the first step in our data analysis, a model of empty MCM‐41 was refined against experimental neutron scattering data, and then filled with the number of benzene molecules corresponding to the atomic density of the bulk liquid, that is, 0.0809 atoms Å^−3^.^17^ Figure 1 shows the total structure factor obtained from the EPSR simulation filled with benzene‐*d_6_* compared with experimental data. In general, the model represents the measured nanoscale structure of the sample well, with good agreement between experimental and simulated total structure factors found in the Bragg scattering arising from the material. These features are in the correct positions, but intensities are not perfectly matched due to the fact that the simulation assumes an idealized repeating crystal, and only artificial broadening of the peaks is applied. Moreover, only one size of a pore is used in the model, whereas in the real system a narrow but significant distribution of pore sizes is observed, and some pores in the real system are likely to be under‐filled or completely blocked, and thus not accessible to benzene molecules. Nevertheless the simulated data gives confidence in the model, and these small discrepancies do not influence the local ordering of the liquid modelled in the pores which is predominantly reflected in the structural signal found in the higher‐*Q* range. In this higher‐*Q* region, the molecular interactions are well modelled as is evident from the excellent agreement with the experimental data.

From the refined atomistic model, we begin by calculating the radial distribution function for the centers of the geometry of the benzene molecules and compare this with the function obtained for the bulk liquid (Figure 2).^17^ The first peak maximum in the two systems is found to be at 6.0 and 5.9 Å for the confined and bulk conditions, respectively, and this 0.1 Å difference is also observed in the most probable placement of the second coordination shell of molecules found in the liquids that is, 10.4 Å for the confined liquid and 10.3 Å for bulk liquid benzene. This observation indicates that surrounding molecules from the first and second coordination shells are organized around the central molecule in a very similar manner in both cases. It should be noted that the radial distribution function for the confined liquid was not corrected for excluded volume effects. The excluded volume is the volume that is inaccessible to other molecules in the system, and in the confined liquids simulations this inaccessible space is occupied by silicon, oxygen and water molecules forming the pore. Effectively this means that the number of surrounding molecules is normalized to the whole simulation box volume, rather than to the pore volume where the molecules can be found. The absence of the excluded volume correction means that the intensities of the peaks are not directly comparable, but the peak profiles are reliable.^18^ A change in the shape of the first coordination shell of the confined liquid when compared to the bulk phase can be noticed with a shoulder at a distance of about 5.5 Å from the central molecule observed for the confined liquid (indicated in Figure 2), and this suggests a difference in the local orientational ordering between the confined and bulk liquid benzene molecules.

**Figure 2:**
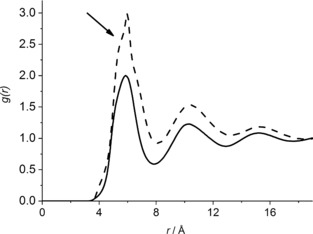
Radial distribution functions between molecular centers for benzene‐*d_6_* confined in MCM‐41 (dashed line). Solid line represents the radial distribution function for the bulk liquid.^17^ The shoulder at 5.5 Å is indicated with an arrow.

The coordination numbers of the closest neighboring molecules show that fewer molecules are found in the first coordination shell for the confined system loaded with 336 benzene molecules (11.6), than are found for the corresponding bulk liquid phase (13.4). This may be due to the large number of surface interactions between the liquid and the silica substrate.

The cylindrical distribution function plotted in Figure 3 a shows the distribution of molecular centers across the pore calculated for a slice of width 4.0 Å, with the pore center located at *r*=0 Å. Benzene‐*d*_6_ molecules occupy four concentric cylindrical shells across the pore radius, with the most organized region found next to the pore wall (indicated in Figure 3 a). This reflects the effect of the interface on the molecular distribution enforcing more ordering in the local environment close to the pore wall. An increase in the function intensity at the pore center is the signature of benzene‐*d_6_* molecules presence in the center of a pore, which is also confirmed by the snapshot of simulation box (see Figure S1 in the Supporting Information) that indicates a uniform distribution of molecules across a pore diameter. In total, four concentric cylindrical coordination shells of benzene are distributed across a pore radius. The higher intensity of the peak in Figure 3 a corresponding to the central layer of molecules, when compared to the remaining peaks, is an artefact caused by counting molecules occurring in a very small volume. This is confirmed by the calculated running coordination number for the slice of width of 4.0 Å. As shown in Figure 3 a, the central concentric cylindrical shell contains about 1 benzene molecule, whereas the shell at distance *r*=5.5 Å from the pore center accommodates 2.3 molecules. The next two concentric cylindrical coordination shells are much more populated than the former two: the shell at distance *r*=10.0 Å from the pore center consists of about 10.2 molecules and the shell next to the pore wall −13.5 benzene molecules.

**Figure 3:**
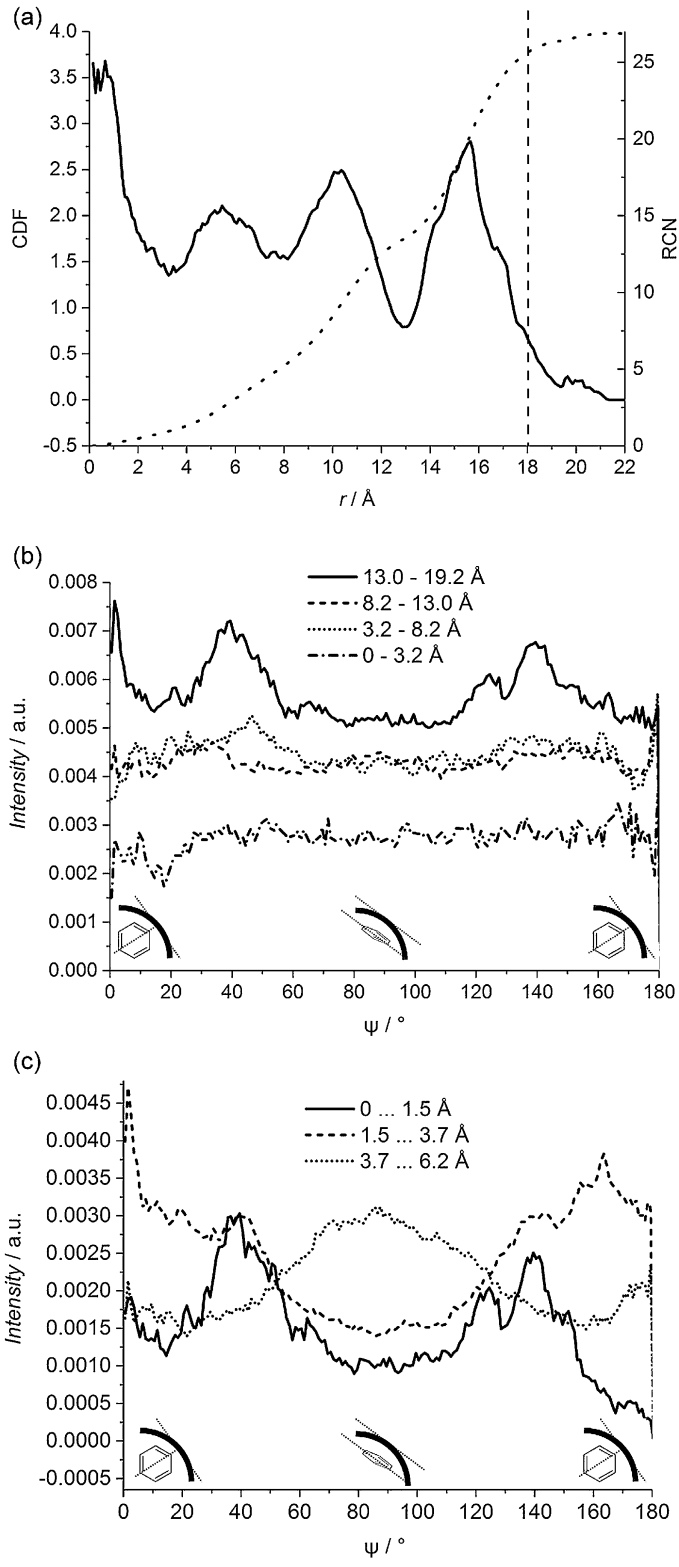
a) Cylindrical distribution function (solid line) across pore calculated for benzene‐*d_6_* confined in MCM‐41. The pore center is at *r*=0 Å, and the pore wall at *r*≈18 Å (dashed line). Dotted line indicates a running coordination number. b) Molecular orientations distribution calculated for each cylindrical concentric shell determined from Figure 3 a, that is, a shell at distance of 0–3.2 Å (dash‐dotted line), 3.2–8.2 Å (dotted line), 8.2–13.0 Å (dashed line) and 13.0–19.2 Å (solid line) from the center of the pore. Distribution of an angle *ψ* between *z*‐axis of a molecule (defined as shown in Figure 4 c) and a vector along the pore is shown. Therefore, *ψ*=90° angle indicates molecules oriented flat to the pore wall, whereas *ψ*=0° and *ψ*=180° angle—molecules pointing with C−H bond towards the pore wall. No offset is used. c) Molecular orientations distribution calculated for different regions within the concentric cylindrical shell that is located close to the pore wall, that is, 0–1.5 Å (solid line), 1.5–3.7 Å (dashed line) and 3.7–6.2 Å (dotted line) from the pore wall. Distribution of an angle *ψ* (defined above) is shown. No offset is used.

The distribution of orientation angle *ψ* between *z*‐axis of a benzene molecule (defined as shown in Figure 4 c) and a vector along the pore axis (Figure 3 b) was calculated for each concentric shell of benzene molecules identified in Figure 3 a. Within this definition *ψ*=90° indicates molecules oriented with the ring plane parallel to the pore wall, whereas *ψ*=0° and *ψ*=180° correspond to molecules oriented with the ring plane perpendicular to the pore wall. In the most central shell of benzene molecules along the pore axis, no significant orientational preference to the pore wall is observed. Like the layer in the center of the pore, the next two layers also do not display any strong orientational preference. The effect of confinement on the resulting preferred orientation of benzene molecules relative to the confining surface appears to be predominantly restricted to the layer in direct contact with the pore wall. This results in a general preference for the molecules adopting a relative orientation where they generally prefer to be canted at an angle of 40° to the silica surface, however it is also interesting to look at the orientational preferences in this contact layer with higher resolution. In order to gauge the impact upon the molecular orientation of benzene molecules by the pore wall, the same distribution of molecular orientations has been calculated with higher distance resolution across the molecular layer that is directly in contact with the confining surface. Three different regions within this cylindrical shell have been selected and the resulting orientation distributions extracted (Figure 3 c). The function reveals that benzene molecules exhibit different orientational preferences as the wall is approached. Molecules that are found within 1.5 Å from the pore wall have a tendency to be canted at an angle of ±40° to perpendicular. In the region that is found between 1.5 and 3.7 Å from the pore wall, the majority of benzene molecules are found with the ring plane oriented preferring to approach a directly perpendicular configuration to the pore wall, albeit over an angular range of perpendicular ±50°. In marked contrast to these configurations, the benzene molecules in the first layer region furthest from interface, in the distance range from 3.7 and 6.2 Å, favor configurations parallel to the pore wall.

**Figure 4:**
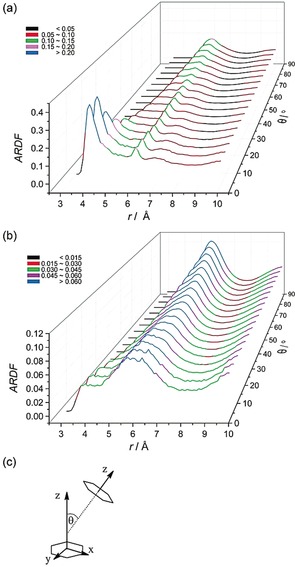
Angular radial distribution function calculated as a function of the angle between the *z*‐axes of the central and surrounding molecules (0<*θ*<90°), which in the studied liquid has been defined as shown in (c). The plot (a) is the function obtained for benzene‐*d_6_* confined in MCM‐41 and (b) for bulk benzene.^17^

Beyond the observed effect of the pore wall upon the preferred molecular orientations, the orientational correlations between the benzene molecules themselves have also been investigated by calculating the angular radial distribution functions (Figure 4), that is, the radial distribution function plotted as a function of the angle *θ* between the *z*‐axes of the central and surrounding molecules (0<*θ*<90°). Parallel molecules are indicated by an angle *θ* between molecular ring planes of 0±10°, whereas perpendicular show a *θ* of 90±10° (axes defined as in Figure 4 c). The function shows a very different trend for surrounding molecule orientations when compared with the function for bulk benzene^17^ (Figure 4 a and b). In bulk liquid benzene, at distances shorter than 4.85 Å, the molecules tend to have a parallel orientation; whereas, at longer distances, perpendicular orientation is preferred (although parallel geometries can still be found). In contrast, for the confined benzene, at the distances shorter than *r*<5.25 Å, the surrounding molecules are mainly oriented in a parallel manner around the central molecule. This preference is much more pronounced than in the bulk phase and suggests that confinement significantly influences this aspect of the structure.

Detailed information concerning the positions occupied by the surrounding benzene molecules can be visualized through the spatial probability densities, Figure 5 (for more details see the Supporting Information). These represent the most probable positions for surrounding molecules within the specified distance from the central molecule and also can be calculated for specific orientations of surrounding molecules (defined as in Figure 4 c). Spatial probability densities for the confined benzene‐*d_6_* are compared with the functions for the bulk liquid benzene.^17^ The cutoff distance used for plotting these functions corresponds to the distances found in the angular radial distribution function for bulk benzene.^17^ No change in the local ordering of surrounding molecules was found at shorter separations in confined benzene, that is, *r*<4.85 Å, where the trend of molecular stacking below and above the ring is preserved (see Figure S2 in the Supporting Information). Similarly, these positions are occupied by confined and bulk benzene also at longer separations (Figure 5 a). Although, at longer distances from the central molecule, the molecules under confinement seem to prefer to occupy positions in front of hydrogen atoms in the plane of the molecule ring, rather than in the middle of C−C bonds as is observed for the bulk liquid. These changes are due to the interactions of parallel molecules (Figure 5 b), and these weaker interactions are modified by confinement while the stronger, namely π‐π interactions causing stacking of molecules above and below the ring, are preserved. Perpendicular molecules show the same most probable positions in both the confined and bulk liquids^17^ (Figure 5 c).

**Figure 5:**
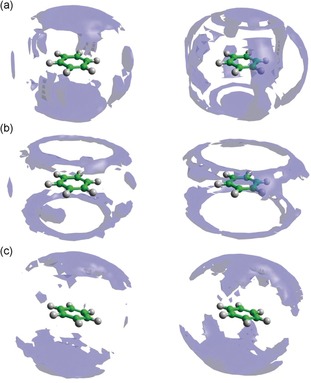
Spatial probability densities for liquid benzene calculated within 4.85–7.90 Å from the central molecule (the range determined from the angular radial distribution function of the bulk liquid^17^). The functions represent: a) the top 12.5 % of all molecules found within *r*=4.85–7.90 Å, b) the top 15 % of parallel molecules only (*θ*=0±10°) and c) the top 15 % of perpendicular molecules only (*θ*=90±10°) with respect to the central molecule. Left‐hand column shows functions for the confined liquid, while those from the bulk liquid are shown in the right‐hand column.^17^

Benzene under confinement exhibits different structural properties than in the corresponding bulk liquid phase, which is evident in the radial distribution functions. Coasne et al. compared these functions for both bulk phase and liquid confined in a cylindrical silica nanopore of diameter 36 Å obtained from grand canonical Monte Carlo simulations.^19^ They concluded that the confined liquid has a liquid‐like structure due to the presence of only short‐range order. Additionally, it was suggested that the confined liquid is less ordered than in the bulk phase due to the interaction with the pore wall. In a molecular dynamics study of benzene confined in carbon nanotubes, even the deformation of the benzene ring is suggested.^20^ Similar concentric cylindrical shells of benzene molecules, as shown herein, were found with Monte Carlo simulations of liquids confined in MCM‐41 with a pore diameter of 32 Å, but the model had no silanol groups in the structure.^21^ According to different Monte Carlo simulation results for MCM‐41, six to seven layers of benzene molecules were found across pores with diameters of 32 and 47 Å,^2^, ^22^ which is in line with the results we have obtained. Some benzene‐*d_6_* molecules enter the pore wall (less than 1.5 % of all molecules), which is due to small irregularities in the surface arising as a result of the refinement procedure.

Molecules adsorbed on the pore wall exhibit a tendency for the orientation with ±40° angle between the ring plane and the pore wall. Moon also suggested that benzene molecules near the pore wall mainly orient their ring planes at a slightly tilted angle with respect to the pore wall, that is, 30° and 150°.^21^ On the contrary, the tendency for molecules to have the rings parallel to the wall was found in grand canonical Monte Carlo and molecular dynamics simulations performed on benzene confined in MCM‐41 with pore diameters of 20, 33 and 36 Å.^2^, ^23^ This tendency was found in our study but for molecules further away from the pore wall than adjacent to the interface. The confinement affects molecule–molecule orientations, and has a local effect, that is, only on molecules in cylindrical shells adjacent to the pore wall. This was also found in the Monte Carlo study on benzene confined in MCM‐41.^21^ Coasne et al. showed that the orientations of benzene molecules adsorbed at the pore wall depend strongly on the silanol group concentration in the system^19^ but this effect has not been investigated in this study.

The obtained structural properties show that the structure of liquid benzene in confinement is markedly different from that found in its corresponding bulk liquid phase. These differences are most visible in the distribution of preferred orientations of surrounding molecules (angular radial distribution functions) and in the favorable positions of parallel molecules around the central molecule (spatial density probability). The distribution of molecules across the pore indicates well‐defined coordination shells formed by the compounds. The fact that confinement tends to enforce a different local ordering between benzene molecules compared to the bulk phase may suggest a different degree of reactivity. This study represents the first step in modelling individual state‐points of the heterogeneously catalyzed liquid‐phase reacting system. To go further with reacting systems, it will be necessary to extend the model of the confined fluid to molecular mixtures, where the relative composition of the fluid will depend on how far along the reaction pathway the system has progressed. Although the information required to perform this task could be extracted from the scattering data alone, it is beneficial if the information on fluid composition can be obtained by complementary in situ characterization techniques such as NMR spectroscopy. This is part of an ongoing project.

## Conflict of interest

The authors declare no conflict of interest.

## Supporting information

As a service to our authors and readers, this journal provides supporting information supplied by the authors. Such materials are peer reviewed and may be re‐organized for online delivery, but are not copy‐edited or typeset. Technical support issues arising from supporting information (other than missing files) should be addressed to the authors.

Supplementary
