# Supplementary material for: Confinement Effects on the Benzene Orientational Structure
Source: Angew Chem Int Ed Engl. 2018 Mar 13;57(17):4565–70. doi: 10.1002/anie.201713115 (PMC6099463; doi:10.1002/anie.201713115)
Supplement: Supplementary file 1 — Supplementary [file ANIE-57-4565-s001.pdf]

Angewandte Chemie
Eine Zeitschrift der Gesellschaft Deutscher Chemiker

Supporting Information

Confinement Effects on the Benzene Orientational Structure

Marta Falkowska, Daniel T. Bowron, Haresh Manyar, Tristan G. A. Youngs,* and Christopher Hardacre**

anie_201713115_sm_miscellaneous_information.pdf

Experimental Details

The surface area of MCM-41 reaches values almost up to 1000 $m^2\ g^{-1}$ due to its inherent nanoporosity and this allows absorption of fluids into the material using capillary condensation techniques. In this study this was achieved by exposure of a previously evacuated catalyst sample to benzene-d_6 vapor at 298 K. This sample loading method is minimally disruptive to the physical packing of the powder sample, which is important for sample-condition sensitive, experimental methods such as neutron diffraction. Furthermore, this procedure minimizes the chance of excess liquid surrounding the powder grains. The benzene absorbed by the material is almost entirely inside the pores, although it is accepted that a small, and for our purposes negligible, amount of liquid may be deposited on the rough external surface of catalyst grains and close points of contact between support grains.

Total neutron scattering data were collected on the Near and InterMediate Range Order Diffractometer (NIMROD)[R1] at the ISIS Pulsed Neutron and Muon Source at Rutherford Appleton Laboratory, UK. The instrument is able to collect data over a Q-range of 0.01 to 50 $Å^{-1}$, which corresponds to the elucidation of atomic pair correlations over length scales ranging from <1 to >300 Å. Raw data were corrected using the GudrunN software.[R2] The analysis of the scattering data consists of constructing an atomistic model of an unfilled catalyst consistent with the experimental data (not shown here) and subsequently filling it with benzene-d_6. Both models were refined against experimental data – total neutron scattering patterns collected for the evacuated and benzene-filled catalyst, respectively. It should be pointed out that in both the dry and filled MCM-41 models, the presence of platinum sites in the substrate is neglected and only silicon and oxygen atoms are used. This is because the low metal loading (3 wt% Pt/MCM-41) makes the structural contribution of the Pt nanoparticles to the total neutron scattering signal too small for detection. Silanol groups present in the support structure are mimicked by water molecules (silicon to hydrogen atom ratio of 0.25). It has been found that a model with a 46.53 x 46.53 x 50 Å monoclinic unit cell (α = 60°) and density of 0.031136 atoms $Å^{-3}$ shows the best agreement with the experimental data. A pore with radius of 18 Å was used, which is consistent with adsorption isotherms results (Brunauer–Emmett–Teller method). Structural properties were calculated using custom analysis codes.[R3]

The process of filling the pores of the catalyst with benzene-d_6 was monitored via the overall level of the neutron scattering signal, as well as through changes in the Bragg peak intensities which are modified as the neutron scattering contrast between the pore walls and the pore contents changes (*i.e*. as the pore fills with benzene). The capillary filling process finished after ~30 min as indicated by the stabilization of the features noted above. Deuterated benzene was utilized in the experiment to ensure the best structural signal to background ratio in the neutron diffraction measurement. The analysis of the Bragg peak intensities of filled and evacuated catalyst, which are related to the squares of the contrast (expressed by differences in scattering length densities), led to the conclusion that pores were filled almost completely, with the amount of benzene-d_6 corresponding to 99.5% of bulk density.

[R1] D. T. Bowron, A. K. Soper, K. Jones, S. Ansell, S. Birch, J. Norris, L. Perrott, D. Riedel, N. J. Rhodes, A. Botti, M.-A. Ricci, F. Grazzi, M. Zoppi, *Rev. Sci. Inst.* **81**, 033905 (2010) . *Compt. Chem.* **2015**, 36, 901-906;

[R2] A. K. Soper, Rutherford Appleton Laboratory Technical Report **2011** RAL-TR-2011-013.

[R3] T. G. A. Youngs, dlputils, version 1.4.5; http://www.projectaten.net/dlputils (accessed 12 December, 2017)

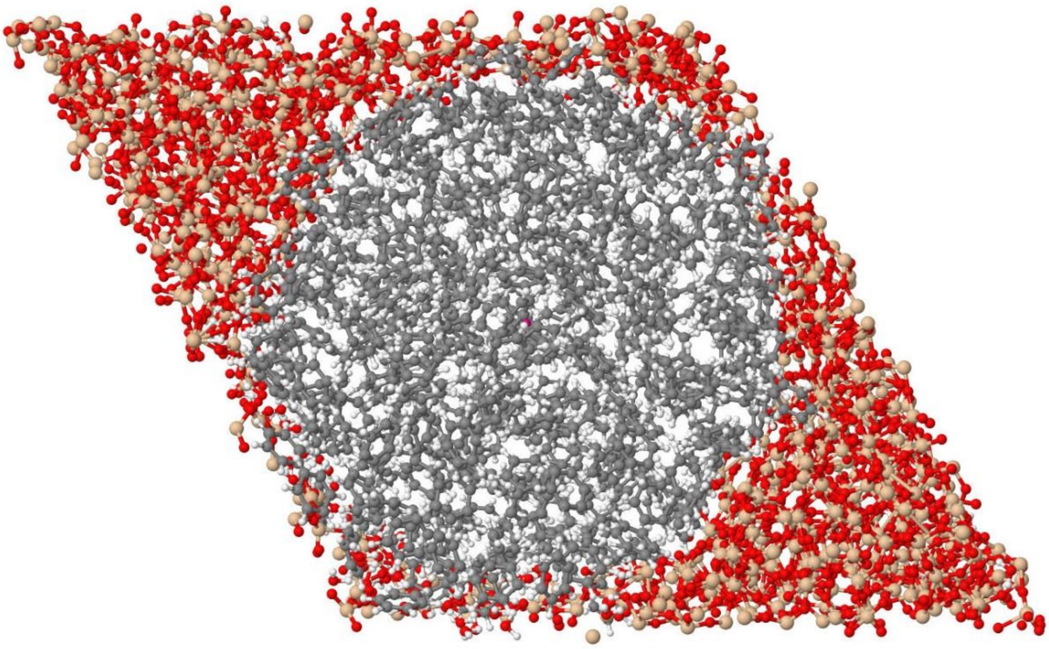

Figure S1. A snapshot of EPSR-simulation box filled with 336 benzene-d_6 molecules. Benzene molecules are represented by grey (carbon) and white (hydrogen) spheres, whereas silica by red (oxygen) and beige (silicon) spheres.

Spatial probability densities represent the most probable positions of one species around another. A set of axes is chosen which represents the orientation of a central molecule in the simulation box. A 3-dimensional histogram is calculated for the positions of all other molecules present in the box, and averaged over all possible central molecules, and so a 3-dimensional equivalent of the radial distribution function is obtained. Blue patches shown in Figure 5 represent the places where neighboring molecules are found with the highest probability.

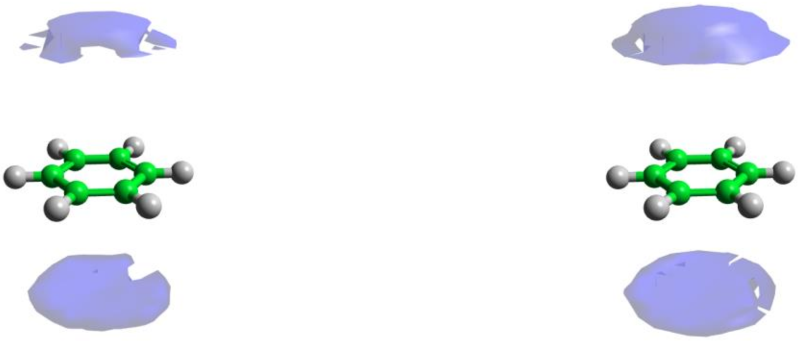

Figure S2. Spatial probability densities for liquid benzene calculated within 0-4.85 Å from the central molecule (the range determined from the angular radial distribution function for bulk liquid[17]). The functions represent the top 25 % of all molecules found within the specified distance range from the central molecule. Left-hand column shows functions for the confined liquid, while those from the bulk liquid are shown in the right-hand column.[17]
